# Voltage-Mode Multifunction Biquadratic Filter with One Input and Six Outputs Using Two ICCIIs

**DOI:** 10.1155/2014/432570

**Published:** 2014-05-08

**Authors:** Hua-Pin Chen

**Affiliations:** Department of Electronic Engineering, Ming Chi University of Technology, Taishan, Taiwan

## Abstract

A novel voltage-mode multifunction biquadratic filter with one input and six outputs is presented. The proposed circuit can realize inverting and noninverting low-pass, bandpass, and high-pass filters, simultaneously, by using two inverting second-generation current conveyors (ICCIIs), two grounded capacitors, and four resistors. Moreover, the proposed circuit offers the following attractive advantages: no requirements for component matching conditions, the use of only grounded capacitors, and low active and passive sensitivities. HSPICE and MATLAB simulations results are provided to demonstrate the theoretical analysis.

## 1. Introduction

There has been an increasing interest in the design of multifunction biquadratic filters. This type of filters can be used in some systems that employ more than one filter function. Filters are widely used in many communications, signal processing, automatic control, and instrumentation systems. For example, two system block diagrams of the receiver/transmitter part of a global system for mobile cellular telephone and crossover network used in a three-way high-fidelity loudspeaker are introduced in [1, 2]. Circuits simultaneously realizing low-pass, bandpass, and high-pass filters find applications in crossover networks used in three-way high-fidelity loudspeakers and touch-tone telephone systems [2]. In analog circuit design, current-mode active devices have been increasingly used to realize active filters and sinusoidal oscillators [1–21]. These current-mode active devices exhibit higher accuracy, wider frequency response, larger dynamic range, greater linearity, and lower power consumption over the operational amplifier-based circuits. As a result, numerous voltage-mode multifunction biquadratic filters using different types of current-mode active devices have received significant attention in technical literature [2–10]. However, none of these filters simultaneously realizes both inverting and noninverting type of low-pass, bandpass, and high-pass responses. The inverting second-generation current conveyor (ICCII) was proposed by Awad and Soliman [11] and has been found useful in many applications [12–15]. An interesting ICCII-based voltage-mode multifunction biquadratic filter with single input and six outputs employing two grounded capacitors and four resistors is proposed [16]. This filter simultaneously realizes inverting and noninverting low-pass, bandpass, and high-pass filtering responses in the same configuration. It also does not require passive element matching conditions and has low active and passive sensitivity performances. However, the *X* ports of the ICCIIs in this circuit design are connected to capacitors and cannot absorb the parasitic capacitances at the *Z* or/and *Y* terminals of the ICCIIs. Because the ICCII has a nonnegligible output parasitic resistance on port *X* (*R*_*X*_), when the *X* port of ICCII is loaded by a capacitor, it leads to improper transfer functions. Due to the effect of this parasitic resistance *R*_*X*_ at the *X* port of ICCII, the circuits with *X* port loaded by a capacitor do not exhibit good performance at high frequency [15–17].

In this paper, a new voltage-mode multifunction biquadratic filter with single input and six outputs is presented. The proposed circuit employs two ICCIIs, two grounded capacitors, and four resistors. The inverting and noninverting low-pass, bandpass, and high-pass filtering responses can be obtained simultaneously. The proposed circuit does not require passive element matching conditions and has low active and passive sensitivity performances. With respect to the previous ICCII-based inverting and noninverting low-pass, bandpass, and high-pass multifunction biquadratic filter in [16], the *X* ports of the ICCIIs in the proposed circuit are connected to resistors. This design offers the feature of a direct incorporation of the parasitic resistance at the *X* ports of the ICCIIs *R*_*X*_, as a part of the main resistance. Moreover, the two external capacitors are grounded and can absorb the parasitic capacitances at the *Z* or/and *Y* terminals of the ICCIIs. To the best of the author's knowledge, none of the previous voltage-mode multifunction biquadratic filters employing only two active components, however, are able to realize inverting and noninverting low-pass, bandpass, and high-pass filters simultaneously without imposing component choice except the circuit reported in [16]; this circuit on the other hand and the two capacitors are connected to the *X* terminals of the ICCII which limits the operating frequency of the circuit [17].

## 2. Proposed Circuit

**Figure 1:**
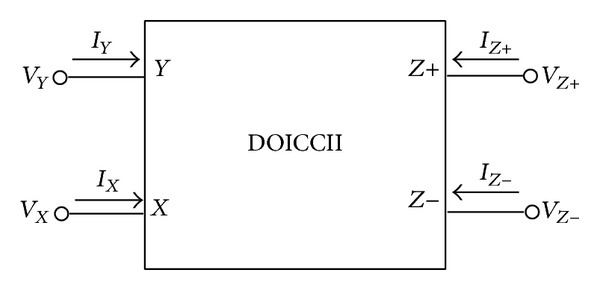
Schematic symbol of DOICCII.

Basically, the port relations of an ideal dual-output ICCII (DOICCII), shown in Figure 1, can be given by the matrix equation:

(1) $$\begin{array}{c} \left[\begin{array}{c} I_{Y}\\ V_{X}\\ I_{Z+}\\ I_{Z-} \end{array}\right]=\left[\begin{array}{cccc} 0 & 0 & 0 & 0\\ -1 & 0 & 0 & 0\\ 0 & 1 & 0 & 0\\ 0 & -1 & 0 & 0 \end{array}\right]\left[\begin{array}{c} V_{Y}\\ I_{X}\\ V_{Z+}\\ V_{Z-} \end{array}\right]. \end{array}$$

**Figure 2:**
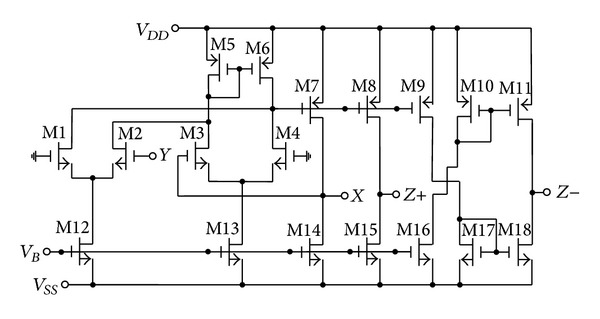
The CMOS implementation of DOICCII.

**Figure 3:**
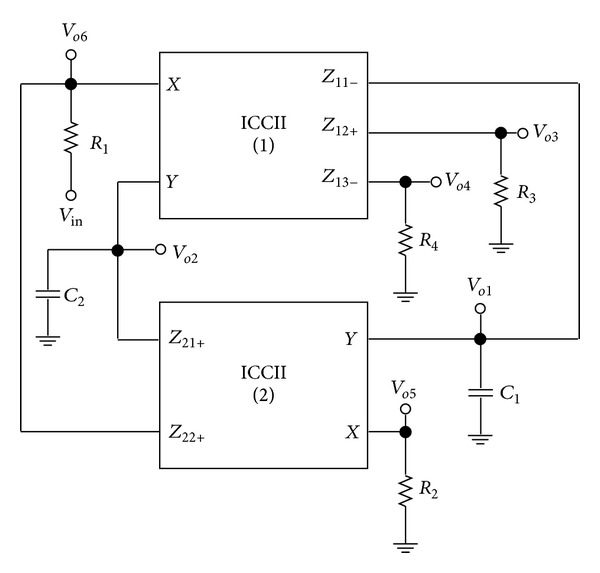
The proposed ICCII-based voltage-mode inverting and noninverting low-pass, bandpass, and high-pass filter.

It is considered to be a special case from the DVCC with single *Y* input only [13]. Various methods can be used to implement CMOS DOICCII. One possible implementation of the DOICCII is shown in Figure 2. The multiple current outputs can be easily implemented by simply adding output branches. The proposed configuration is shown in Figure 3. It employs two multioutput ICCIIs, two grounded capacitors, and four resistors. The use of grounded capacitors is attractive from a monolithic integration point of view because grounded capacitor circuits can compensate for the stray capacitances at their nodes [6]. Because each *X* terminal of the ICCII in the proposed circuit of Figure 3 is directly connected to an external resistor, the effect of parasitic resistance *R*_*X*_ can easily be absorbed as a part of the main resistance. Straightforwardly analyzing the filter in Figure 3, the following six filter voltage transfer functions can be simultaneously derived as

(2) Vo1Vin=sC2R2s2C1C2R1R2+sC2R1+1,Vo2Vin=−1s2C1C2R1R2+sC2R1+1,Vo3Vin=−(R3R1)s2C1C2R1R2s2C1C2R1R2+sC2R1+1,Vo4Vin=(R4R1)s2C1C2R1R2s2C1C2R1R2+sC2R1+1,Vo5Vin=−sC2R2s2C1C2R1R2+sC2R1+1,Vo6Vin=1s2C1C2R1R2+sC2R1+1.

Clearly, the filter simultaneously realizes second order inverting and non-inverting low-pass, bandpass, and high-pass filtering responses without requiring any passive component matching condition. Since the input impedance of the proposed circuit is not high, voltage follower is needed while cascading the proposed circuit to the next stages. It is also to be noted that the output terminals of the proposed circuit are not in low-output impedances. Voltage followers are needed for the proposed circuit to drive low impedance loads or to be directly connected to the next stages.

The resonance angular frequency (*ω*_*o*_), the quality factor (*Q*), and bandwidth (BW) are given by

(3) ωo=1R1R2C1C2,Q=R2C1R1C2,BW=ωoQ=1R1C1.

This shows that the proposed filter enjoys orthogonal control of *ω*_*o*_ and BW by tuning grounded resistor *R*_2_ for *ω*_*o*_ first and then resistor *R*_1_ for BW without disturbing parameter *ω*_*o*_, but not vice versa. However, the technique to obtain the noninteractive filter parameter control can be suggested as follows. For the fix-valued capacitors, the *ω*_*o*_ can be adjusted arbitrarily without disturbing *Q* by simultaneously changing *R*_1_ and *R*_2_ and keeping the quotient *R*_2_/*R*_1_ constant. On the other hand, the parameter *Q* can be tuned arbitrarily without disturbing *ω*_*o*_ by increasing *R*_1_ and decreasing *R*_2_ (or decreasing *R*_1_ and increasing *R*_2_) simultaneously, while keeping the produce *R*_1_ *R*_2_ constant.

By taking into account the nonidealities of DOICCII, the relationship of the terminal voltages and currents can be rewritten as *V*_*X*_ = −*βV*_*Y*_, *I*_*Z*+_ = *αI*_*X*_, and *I*_*Z*−_ = −*ηI*_*X*_, where *β* = 1 − *ε*_*v*_ and *ε*_*v*_ (|*ε*_*v*_| ≪ 1) denotes the voltage tracking error from *Y* terminal to *X* terminal of the DOICCII, *α* = 1 − *ε*_*αi*_ and *ε*_*αi*_ (|*ε*_*αi*_| ≪ 1) is the current tracking error from *X* terminal to *Z*+ terminal of the DOICCII, and *η* = 1 − *ε*_*ηi*_ and *ε*_*ηi*_ (|*ε*_*ηi*_| ≪ 1) is the current tracking error from *X* terminal to *Z* terminal of the DOICCII. Reanalysis of the proposed circuit in Figure 3 yields the denominator of the nonideal voltage transfer functions as follows:

(4) $$\begin{array}{c} D\left(s\right)=s^{2}C_{1}C_{2}R_{1}R_{2}+\eta_{11}\alpha_{22}\beta_{2}sC_{2}R_{1}+\eta_{11}\alpha_{21}\beta_{1}\beta_{2}. \end{array}$$

The filter parameters for the nonideal *ω*_*o*_ and *Q* are obtained as

(5) ωo=η11α21β1β2R1R2C1C2,Q=1α22α21β1R2C1η11β2R1C2.

The active and passive sensitivities of *ω*_*o*_ and *Q* of the proposed filter are

(6) $$\begin{array}{c} S_{\eta_{11},\alpha_{21},\beta_{1},\beta_{2}}^{\omega_{o}}=-S_{R_{1},R_{2},C_{1},C_{2}}^{\omega_{o}}=\frac{1}{2},\\ S_{\alpha_{21},\beta_{1}}^{Q}=-S_{\eta_{11},\beta_{2}}^{Q}=S_{R_{2},C_{1}}^{Q}=-S_{R_{1},C_{2}}^{Q}=\frac{1}{2},\\ S_{\alpha_{22}}^{Q}=-1. \end{array}$$

The active and passive of the sensitivities remain less than or equal to one in magnitude.

## 3. Influence of Parasitic Elements

**Figure 4:**
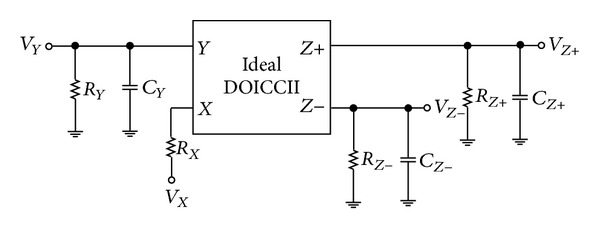
The nonideal DOICCII model.

A nonideal DOICCII model is shown in Figure 4. It is shown that the real DOICCII has a low-value parasitic serial resistance at port *X* (*R*_*X*_) and high output impedances at ports *Y* (*R*_*Y*_//*C*_*Y*_) and *Z* (*R*_*Z*_//*C*_*Z*_), respectively. Because each *X* terminal of the ICCII in the proposed circuit of Figure 3 is directly connected to an external resistor, the effect of parasitic resistance *R*_*X*_ can easily be absorbed as a part of the main resistance. It is further noted that the proposed circuit of Figure 3 employs external grounded capacitors *C*_1_ and *C*_2_ parallel connecting at the ports *Y* and *Z* of ICCIIs, respectively. The effects of parasitic capacitances can also be absorbed. Hence, in practical ICCIIs, the external resistors can be chosen to be much smaller than that of the parasitic resistors at the *Y* and *Z* terminals of ICCIIs and much greater than the parasitic resistors at the *X* terminals of ICCIIs, that is, *R*_*X*_ ≪ *R*_1_, *R*_2_ ≪ *R*_*Y*_, *R*_*Z*_. The external grounded capacitors *C*_1_ and *C*_2_ can be chosen to be much greater than the parasitic capacitances at the *Y* and *Z* terminals of ICCIIs, that is *C*_*Y*_, *C*_*Z*_ ≪ *C*_1_, *C*_2_. On reanalyzing the proposed voltage-mode filter, taking into account the above parasitic effects, the characteristic equation of Figure 3 becomes

(7) D(s)=s2+s1R2′C1′(1+R2′R1p+R2′C1′R2pC2′)+1R1′R2′C1′C2′(1+R1′R2p+R1′R2′R1pR2p),

where *C*_1_′ = *C*_1_ + *C*_*Y*2_ + *C*_*Z*11_, *C*_2_′ = *C*_2_ + *C*_*Y*1_ + *C*_*Z*21_, *R*_1*p*_ = *R*_*Y*2_//*R*_*Z*11_, *R*_2*p*_ = *R*_*Y*1_//*R*_*Z*21_, *R*_1_′ = *R*_*X*1_ + *R*_1_, and *R*_2_′ = *R*_*X*2_ + *R*_2_.

Assuming that max⁡(*R*_1_′, *R*_2_′)≪(*R*_1*p*_, *R*_2*p*_) and *C*_1_′ ≤ *C*_2_′, the effect of the parasitic impedance effects in (7) can be reduced. Under these conditions, the parameters *ω*_*o*_ and *Q* are changed to

(8) ωo=1R1′R2′C1′C2′,Q=R2′C1′R1′C2′.

It is to be noted that the resistor of the proposed filter is connected to the *X* terminals of multioutput ICCIIs and the capacitor of the proposed filter is connected to the *Y* and *Z* terminals. Thus, all the loading effects of the parasitics can be accommodated.

## 4. Simulation Results

**Figure 5:**
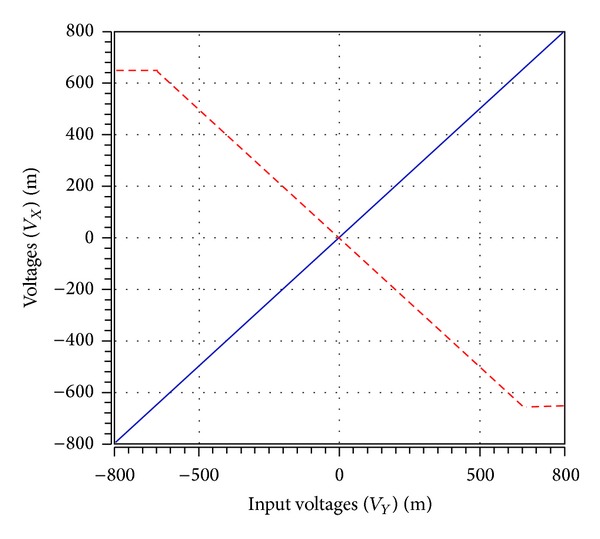
The DC characteristic between the *Y* (blue line) and *X* (red line) terminals voltages of the ICCII.

**Figure 6:**
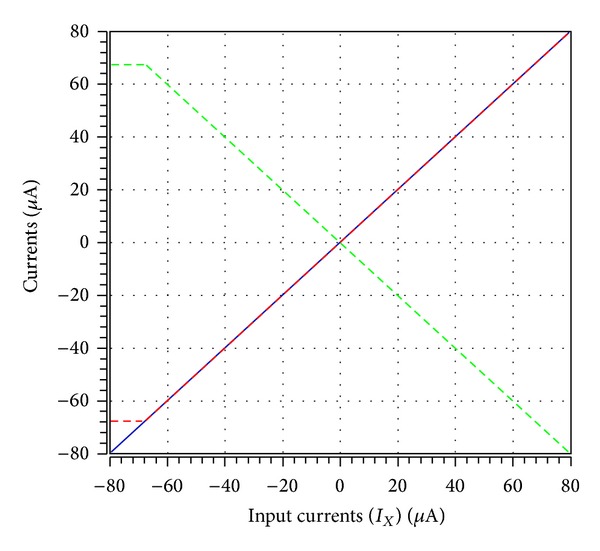
The DC characteristic between the *X* (blue line), *Z*+ (red line), and *Z*− (green line) terminals currents of the ICCII.

**Figure 7:**
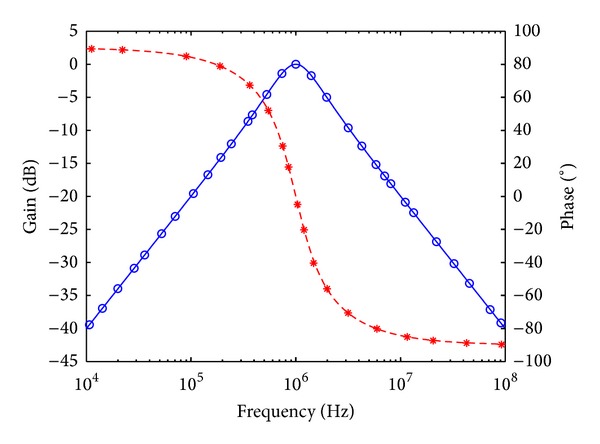
Simulated gain-frequency and phase-frequency responses of noninverting bandpass filter at *V*_*o*1_ (∘: simulated gain; ∗: simulated phase; — and ---: theoretical curves).

**Figure 8:**
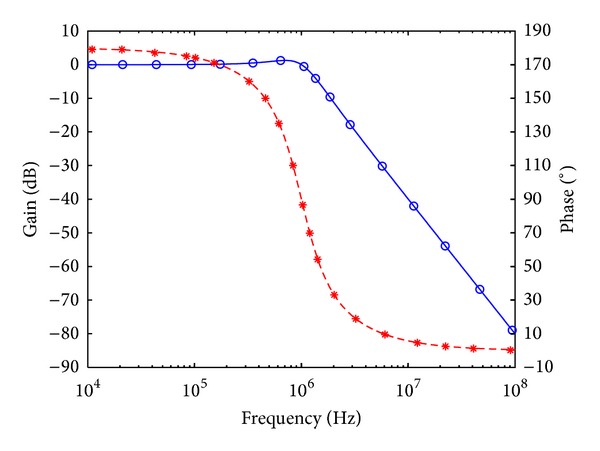
Simulated gain-frequency and phase-frequency responses of inverting low-pass filter at *V*_*o*2_ (∘: simulated gain; ∗: simulated phase; — and ---: theoretical curves).

**Figure 9:**
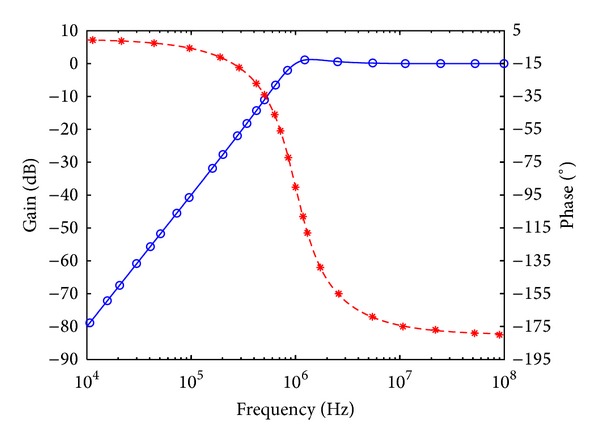
Simulated gain-frequency and phase-frequency responses of inverting high-pass filter at *V*_*o*3_ (∘: simulated gain; ∗: simulated phase; — and ---: theoretical curves).

**Figure 10:**
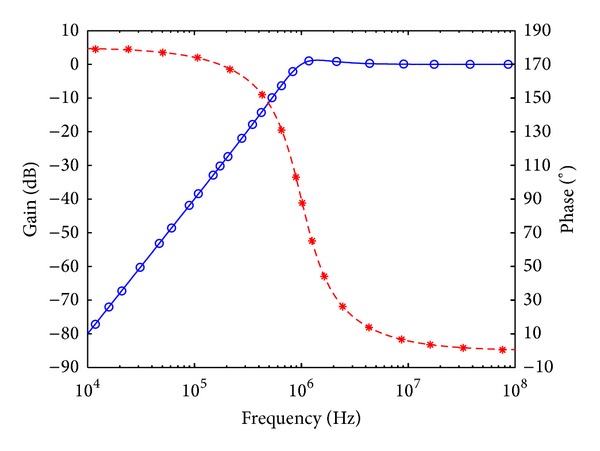
Simulated gain-frequency and phase-frequency responses of noninverting high-pass filter at *V*_*o*4_ (∘: simulated gain; ∗: simulated phase; — and ---: theoretical curves).

**Figure 11:**
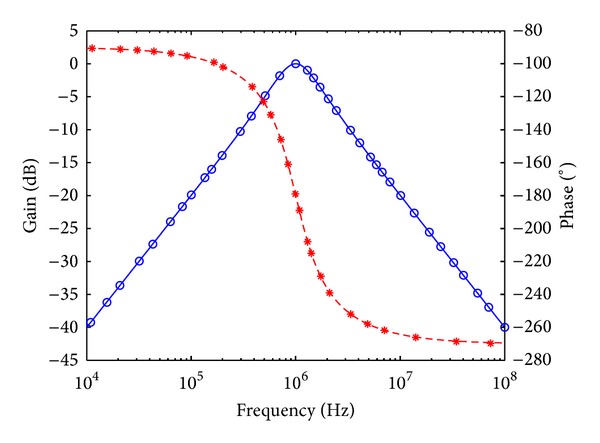
Simulated gain-frequency and phase-frequency responses of inverting bandpass filter at *V*_*o*5_ (∘: simulated gain; ∗: simulated phase; — and ---: theoretical curves).

**Figure 12:**
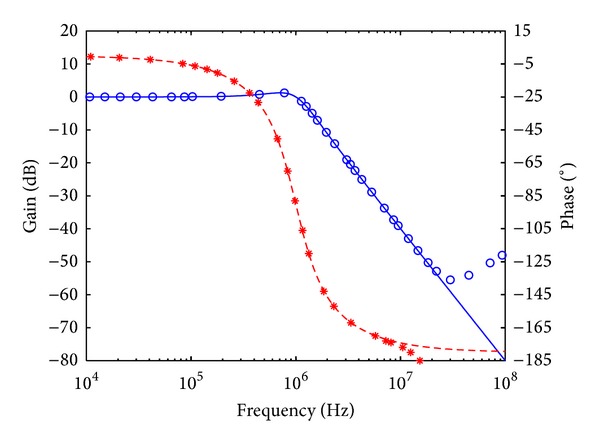
Simulated gain-frequency and phase-frequency responses of noninverting low-pass filter at *V*_*o*6_ (∘: simulated gain; ∗: simulated phase; — and ---: theoretical curves).

**Figure 13:**
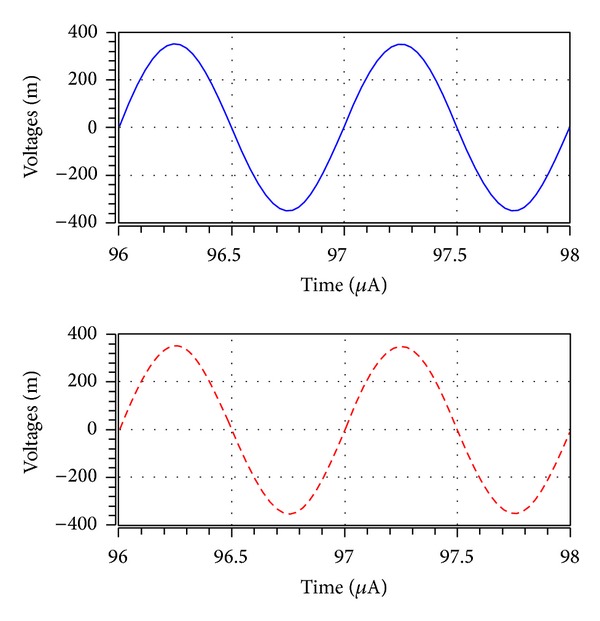
The input (blue line) and output (red line) waveforms of noninverting bandpass filter at *V*_*o*1_ designed with *R*_1_ = *R*_2_ = *R*_3_ = *R*_4_ = 10 kΩ, *C*_1_ = *C*_2_ = 15.9 pF, for a 1 MHz sinusoidal input voltage of 0.7 V (peak to peak).

**Figure 14:**
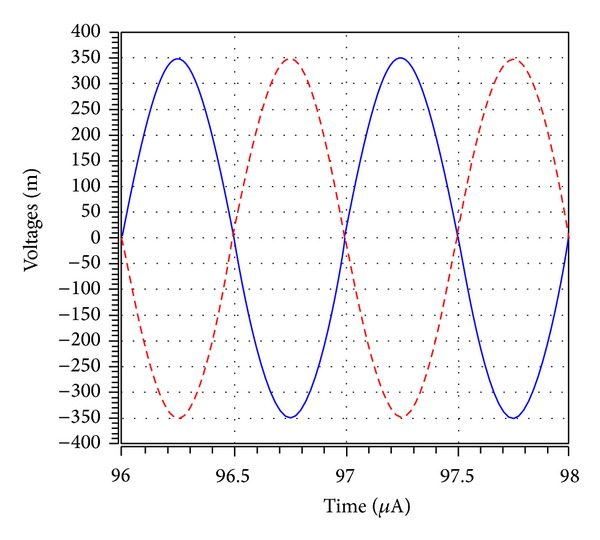
The input (blue line) and output (red line) waveforms of inverting bandpass filter at *V*_*o*5_ designed with *R*_1_ = *R*_2_ = *R*_3_ = *R*_4_ = 10 kΩ, *C*_1_ = *C*_2_ = 15.9 pF, for a 1 MHz sinusoidal input voltage of 0.7 V (peak to peak).

**Figure 15:**
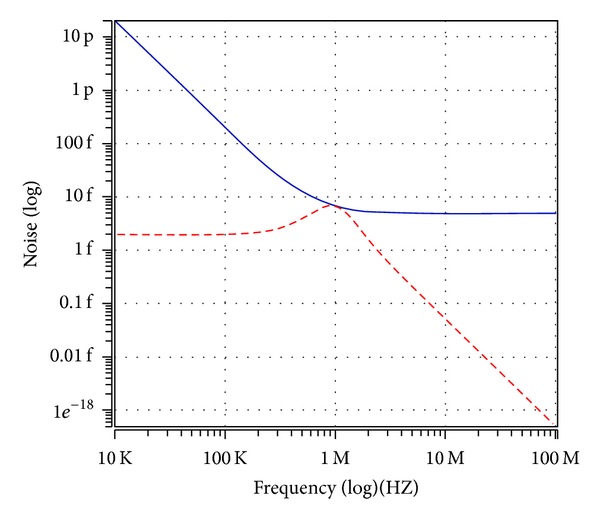
Equivalent input (blue line) and output (red line) noise of BP filter versus frequency.

To verify the theoretical analysis, HSPICE simulations were carried out to demonstrate the feasibility of the proposed circuit. Based on TSMC 0.18 *μ*m CMOS process, Figure 3 is simulated by using Figure 2 for DOICCII. The multiple current outputs of the ICCII are easily obtained by applying current replicas. The supply voltages and biasing voltage are given by *V*_*DD*_ = −*V*_*SS*_ = 0.9 V, and *V*_*B*_ = −0.5 V, respectively. The dimensions of MOS transistors used in implementation of the DOICCII were given in Table 1. The DC characteristic between the *Y* and *X* terminals voltages is shown in Figure 5. The output voltage *V*_*X*_ at *X* terminal is the inversion of the input voltage source *V*_*Y*_ at *Y* terminal. The linearity range extends from −0.65 V to 0.65 V. The DC characteristic between the *X* and *Z* terminals' currents is shown in Figure 6 by connecting a floating input current source *I*_*X*_ at *X* terminal, while the voltage across *Y* terminal is set to zero. The linearity range extends from −67.3 *μ*A to 80 *μ*A. The passive component values of Figure 3 were chosen as *C*_1_ = *C*_2_ = 15.9 pF, and *R*_1_ = *R*_2_ = *R*_3_ = *R*_4_ = 10 kΩ, leading to a center frequency of *f*_*o*_ = 1 MHz, and quality factor *Q* = 1. Figures 7, 8, 9, 10, 11, and 12 show the simulated results of noninverting bandpass (*V*_*o*1_), inverting low-pass (*V*_*o*2_), inverting high-pass (*V*_*o*3_), noninverting high-pass (*V*_*o*4_), inverting bandpass (*V*_*o*5_), and noninverting low-pass (*V*_*o*6_) frequency responses, respectively. The *X* terminal of the ICCII(1) is connected to *Z*_22_+ terminal of the ICCII(2); hence, the parasitic capacitance (*C*_*Z*22_) and resistance (*R*_*Z*22_) affect the high frequency responses. This can explain why Figure 12 has nonideal gain and phase responses. The large signal behavior of the circuit in Figure 3 is also investigated.  Figure 13 shows the input and output signals of noninverting bandpass response at *V*_*o*1_ output terminal. It is observed that 1 MHz with 0.7 V peak to peak input voltage signal levels are possible without signification distortion.  Figure 14 shows the input and output signals of inverting bandpass response at *V*_*o*5_ output terminal. It is also observed that 1 MHz with 0.7 V peak to peak input voltage signal levels is possible without signification distortion. The noise behaviour of the filter was simulated using the INOISE and ONOISE statements.  Figure 15 shows the simulated input and output noise amplitude responses for the noninverting bandpass filter with INOISE and ONOISE. Equivalent input and output noises against frequency are given for the noninverting bandpass response in Table 2. The total equivalent input and output noise voltages were 827.09 *μ*V/$\sqrt{\text{Hz}}$ and 103.36 *μ*V/$\sqrt{\text{Hz}}$, respectively. The output noise was extremely small and did not affect the output signal. The total power dissipation is found to be 0.834 mW. Note that simulation results demonstrated in Figures 7–14 agree quite well with the theoretical ones as expected. Nonetheless, the difference between the theoretical and simulated responses mainly stems from the parasitic impedance effects and nonideal gains of ICCIIs.

## 5. Conclusions

In this paper, a novel voltage-mode multifunction biquadratic filter is proposed. The proposed circuit offers several advantages, such as no requirements for component matching conditions, the simultaneous realization of inverting and noninverting low-pass, bandpass, and high-pass responses from the same configuration, the use of only grounded capacitors, and low active and passive sensitivity performances. The proposed circuit has the same advantages reported by [16] using two ICCIIs, two grounded capacitors, and four resistors. Moreover, the proposed circuit has one more important advantage of direct incorporation of the parasitic resistance at the *X* terminal of the ICCII as a part of the main resistance. The two external capacitors are grounded and can absorb the parasitic capacitances at the *Z* or/and *Y* terminals of the ICCIIs. HSPICE simulations, using TSMC 0.18 *μ*m CMOS process technology and supply voltages ±0.9 V, confirm the theoretical predictions.

## Figures and Tables

**Table 1:** Aspect ratios of the MOS in Figure 2.

Transistors	*L* (*μ*m)	*W* (*μ*m)
M1–M4	0.35	8.75
M5–M11	0.18	17.5
M12–M18	0.18	8.75

**Table 2:** Input and output noises against frequency for noninverting bandpass response.

Frequency (Hz)	Noise	
	Input noise $\text{V}/\sqrt{\text{Hz}}$	Output noise $\text{V}/\sqrt{\text{Hz}}$
10 × 10^3^	4.368 × 10^−6^	43.643 × 10^−9^
25.118 × 10^3^	1.74 × 10^−6^	43.684 × 10^−9^
63.095 × 10^3^	695.784 × 10^−9^	43.946 × 10^−9^
100 × 10^3^	442.379 × 10^−9^	44.416 × 10^−9^
158.489 × 10^3^	284.392 × 10^−9^	45.591 × 10^−9^
251.188 × 10^3^	187.531 × 10^−9^	48.514 × 10^−9^
398.107 × 10^3^	130.269 × 10^−9^	55.649 × 10^−9^
630.957 × 10^3^	98.618 × 10^−9^	71.283 × 10^−9^
1.584 × 10^6^	75.453 × 10^−9^	54.658 × 10^−9^
2.511 × 10^6^	72.36 × 10^−9^	30.98 × 10^−9^
3.981 × 10^6^	71.092 × 10^−9^	18.43 × 10^−9^
6.309 × 10^6^	70.58 × 10^−9^	11.337 × 10^−9^
10 × 10^6^	70.376 × 10^−9^	7.080 × 10^−9^
15.848 × 10^6^	70.294 × 10^−9^	4.448 × 10^−9^
25.118 × 10^6^	70.262 × 10^−9^	2.802 × 10^−9^
39.81 × 10^6^	70.249 × 10^−9^	1.767 × 10^−9^
63.095 × 10^6^	70.244 × 10^−9^	1.114 × 10^−9^
100 × 10^6^	70.242 × 10^−9^	703.183 × 10^−12^
